# Transposable Element Expression and Regulation Profile in Gonads of Interspecific Hybrids of *Drosophila* *arizonae* and *Drosophila mojavensis wrigleyi*

**DOI:** 10.3390/cells10123574

**Published:** 2021-12-18

**Authors:** Cecília Artico Banho, Daniel Siqueira Oliveira, Annabelle Haudry, Marie Fablet, Cristina Vieira, Claudia Marcia Aparecida Carareto

**Affiliations:** 1 Institute of Biosciences, Humanities and Exact Sciences, São Paulo State University (Unesp), São José do Rio Preto 15054-000, SP, Brazil; artico.banho@unesp.br (C.A.B.); daniel.siqueira@unesp.br (D.S.O.); 2 Laboratoire de Biométrie et Biologie Evolutive, Université de Lyon, Université Lyon 1, CNRS, UMR 5558, F-69622 Villeurbanne, France; annabelle.haudry@univ-lyon1.fr (A.H.); marie.fablet@univ-lyon1.fr (M.F.); 3 Institut Universitaire de France (IUF), F-75231 Paris, France

**Keywords:** repleta group, hybrids, transposable elements, expression

## Abstract

Interspecific hybridization may lead to sterility and/or inviability through differential expression of genes and transposable elements (TEs). In *Drosophila*, studies have reported massive TE mobilization in hybrids from interspecific crosses of species presenting high divergence times. However, few studies have examined the consequences of TE mobilization upon hybridization in recently diverged species, such as *Drosophila arizonae* and *D. mojavensis.* We have sequenced transcriptomes of *D. arizonae* and the subspecies *D. m. wrigleyi* and their reciprocal hybrids, as well as piRNAs, to analyze the impact of genomic stress on TE regulation. Our results revealed that the differential expression in both gonadal tissues of parental species was similar. Globally, ovaries and testes showed few deregulated TEs compared with both parental lines. Analyses of small RNA data showed that in ovaries, the TE upregulation is likely due to divergence of copies inherited from parental genomes and lack of piRNAs mapping to them. Nevertheless, in testes, the divergent expression of genes associated with chromatin state and piRNA pathway potentially indicates that TE differential expression is related to the divergence of regulatory genes that play a role in modulating transcriptional and post-transcriptional mechanisms.

## 1. Introduction

Transposable elements (TEs) are repetitive DNA sequences that move from one place to another in the genome and between genomes. Because of their ability to mobilize, these elements play an important role in creating genetic variability and, consequently, in genome evolution and adaptation [1,2,3]. TE activation can be induced by environmental stress [4] and/or by genomic shocks, such as hybridization [5,6,7,8]. The disruption of genome stability following hybridization is attributed to the divergence of regulatory sequences and/or to the content of TEs [9]. Several studies have reported the role of TEs on reproductive isolation, which may be due to their ability to modify regulatory networks and gene expression and lead to structural rearrangements [1,10]. The effects of TEs on intraspecific or interspecific hybrids may be beneficial or deleterious, depending on the species. In *Drosophila* intraspecific hybrids, some TEs are responsible for hybrid dysgenesis syndrome, which is characterized by gonadal atrophy and generally affects offspring obtained from crosses in only one direction [11,12,13,14,15,16]. In interspecific hybrids obtained from highly divergent parental species, massive TE expression and mobilization have been observed [7,9,17,18,19,20,21]. This phenomenon was mainly associated with the lack of small interfering RNA from the class of the Piwi-RNA (piRNA) that are able to control TE mobilization and expression, contributing to hybrid sterility to some extent [7,9,19,20,21]. These 23–30 nt small RNAs act in the *Drosophila* germline at post-transcriptional and transcriptional levels together with a complex of Argonaute proteins, which recognize homologous TEs and induce their degradation [22,23,24,25]. More specifically, in the *Drosophila* germline, primary and secondary piRNAs are found. The primary piRNAs are transcribed from genomic regions named piRNAs clusters. These transcripts are processed and loaded into Piwi and Aubergine (Aub) proteins, which load antisense piRNA transcripts. When TEs are transcribed in the germline, the complex Aub-piRNA recognizes the complementary TEs, degrading them through an endonuclease activity. This process originates sense piRNAs, which are loaded by Argonaute3 (AGO3) proteins. The complex AGO3-piRNAs are responsible for degrading antisense TE transcripts. This phenomenon is known as the ping-pong amplification loop, which is highly efficient in silencing transcribed TEs. Together with this process, the complex Piwi-piRNAs acts in the nucleus promoting transcriptional gene silencing [23].

In intraspecific or interspecific crosses, females lacking one or a few TE families in comparison with the male line cannot produce specific piRNAs, which are maternally deposited, and hence, they are unable to silence TE expression [26]. However, in interspecific hybrids, it seems that adaptative divergence of the piRNA pathway genes and divergence in TE copies between parental lines are the main responsible for TE activation [9,20]. The potential to cause negative genetic interactions via TE derepression in hybrids places TEs within the classical Dobzhansky-Muller model [27,28] for the evolution of incompatibilities. Despite several studies that have analyzed the consequences of hybridization regard-ing TE behavior at an intraspecific level and in hybrids from highly divergent species, few studies are showing the role of TEs in the early stages of hybrid incompatibility.

*D. arizonae* and *D. mojavensis* (repleta group, *Drosophila* genus) are sibling species with divergence time estimated in ~1.5 million years [29]. Moreover, they are able to produce hybrids in the laboratory with asymmetrical sterility [30,31,32,33]. Since the genomes are public, the TE contents of these species can be accessed, enabling a study of differential expression in hybrids and both parental lines. This pair of species is an attractive model to study differences in the TE expression in the reproductive organs of hybrids, as well as their regulation by analyzing the piRNA pathway. Two previous studies have analyzed the expression of TEs in hybrids of *D. arizonae* and *D. m. mojavensis* [34,35] but only in the female germline. There is no study in the male germline, neither in parents nor in hybrids.

Here we aimed to test the hypothesis that the rate of TE deregulation in hybrids is associated with the divergence time of the parental species, which can affect TE regulation in female and male gonads. To test this hypothesis, we designed our study to answer three main questions. First, does the expression of TEs differ between parent species and their reciprocal hybrids, as well as between male and female gonads? Second, is the TE deregulation associated with differential expression of piRNAs in ovaries and testes? Third, the differential expression of piRNAs is due to divergence between genes in the piRNA pathway inherited from the parents. Through transcriptome analyses of parental and hybrid female and male gonads, we showed that the extent of TE deregulation in ovaries and testes is very similar. Hybrid ovaries and testes exhibited very few TE deregulated compared with both parental lines. A different abundance of piRNAs associated with these specific deregulated TEs was observed for ovaries and testes. In ovaries, most of the deregulated TEs had lower levels of specific piRNAs when compared with one of the parental lines, suggesting that divergence of TE copies can play an important role in post-transcriptional regulation mechanisms driven by piRNA in females. However, in males, the amount of piRNA mapping to TEs was very similar to parental lines indicating that other factors can have a role in TE regulation. Among these factors, it is noteworthy that several piRNA genes displayed differential expression in testes and could influence post-transcriptional and transcriptional silencing mechanisms.

## 2. Materials and Methods

### 2.1. Drosophila Strains and Crosses

Intraspecific and interspecific reciprocal crosses were performed between *D. arizonae* from Metztitlan, Hidalgo, México (stock number: 15081-1271.17) and *D. m. wrigleyi* from Catalina Island, California, USA (stock number: 15081-1352.22), according to Banho et al. [30]. The hybrid status of the generated offspring was confirmed by performing DNA extraction and PCR to analyze the ribosomal ITS-1 (Internal Transcribed Spacer 1) sequence from the 18S gene region, NCBI Reference Sequence: EU306666.1 [36], before sequencing. The offspring from interspecific crosses were named according to cross direction, H♀m^wri^♂ari and H♀ari♂m^wri^.

### 2.2. RNA Extraction, Library Preparation, and Sequencing and TE Library Construction

RNA extraction, library preparation, and RNA sequencing data were performed as in Banho et al. [30]. The TE library was built based on TE insertions annotated in *D. m. wrigleyi* and *D. arizonae*, publically available (https://www.ncbi.nlm.nih.gov/assembly/GCF_000005175.2 and https://www.ncbi.nlm.nih.gov/assembly/GCF_001654025.1/GCA_001654025.1, accessed on 10 December 2021). The RepeatMasker [37,38] results for such genomes were obtained from NCBI and filtered to remove repeat sequences that are not TEs, classified as Simple repeat, Satellite, Low complexity, tRNA, rRNA, Unknown, and ATCG enriched regions. In addition, we have filtered all TEs insertions shorter than 100 nt. To merge all LTR sequences with their respective LTR element and small fractions of the same DNA element with overlapping positions in the genome, we have used One Code to Find Them All [39] (http://doua.prabi.fr/software/one-code-to-find-them-all, accessed on 10 December 2021). Due to the RepeatMasker misclassification possibility caused by insufficient sequence similarity to define the correct TE family [40], we have minimized the super estimation of family numbers by removing the species names from family classification (e.g., “*Gypsy-1-Dmoj*” = “*Gypsy-1*”). From this, we have annotated 41,546 TEs for *D. mojavensis* and 17,347 for *D. arizonae*, classified into 276 and 249 families, respectively. These manually curated TE classifications were merged into one file containing 58,893 TE sequences, which were classified in 298 families. As a result, our TE library presents 227 TE families shared between *D. arizonae* and *D. m. wrigleyi*, 49 exclusive to *D. m. wrigleyi*, and 22 exclusives to *D. arizonae* genome.

### 2.3. Small RNA Extraction, Library Preparation, and Sequencing

Small RNAs were extracted from the ovaries (70 pairs) and testes (100 pairs) of *D. arizonae*, *D. m. wrigleyi*, and their interspecific hybrids using as described by Grentizinger et al. [41]. Two Illumina libraries were prepared for each sample from 1 to 6 ng of the purified small RNA fraction using the TruSeq Small RNA Library Prep Kit (Illumina, San Diego, CA), and sequencing was performed by GenomEast platform, a member of the ‘France Génomique’ consortium (ANR-10-INBS-0009)”, using Illumina HiSeq 4000 instrument (read length 1 × 50 bases) (Illumina Inc, San Diego, CA, USA). Overall, 729 million reads were obtained, and reads from 23 to 30 nt were retained as piRNAs.

### 2.4. TE and Gene Read Mapping and Differential Expression Analysis

The sequenced transcriptomes were trimmed using UrQt [42] to remove polyA tails (from RNA-Seq reads) and low quality nucleotides, and then the sequence quality was assessed using FastQC software (available at: http://www.bioinformatics.babraham.ac.uk/projects/fastqc, accessed on 10 August 2021). TE expression analyses were performed with the module TEcount from the TEtools pipeline [37], available at https://github.com/l-modolo/TEtools, 10 August 2021. RNA-Seq reads were aligned to our TE library using Bowtie2 [43]. The read count step was computed for each TE family by adding all reads mapped on copies of the same family.

In addition, the parental and hybrid transcriptomes were aligned to all annotated (20,110 mRNAs) coding sequences (CDS) of *D. mojavensis* r1.04 public genome [available at http://flybase.org/, accessed on 10 December 2021] using Kallisto [44] in order to verify the expression of genes regulating the piRNA pathway, according to the same procedure as described in Banho et al. [30]. We have used these data in the differential expression analyses to identify the genes involved in the piRNA pathway.

Differential expression analyses were performed with the R Biocondutor package DESeq2 [45,46] (available at https://bioconductor.org/packages/release/bioc/html/DESeq2.html, accessed on 10 December 2021) using raw read counts to identify differential expression of TEs and genes in the reproductive tissues from the hybrids compared to the parental species. TE families and genes were classified as differentially expressed (DE) when the adjusted p-value (FDR level) was less than 0.01 and a Log2(FoldChange) ≥ |1|.

### 2.5. Genome Assembling and Analysis of Evolutionary Rates

As divergence of genetic sequences is accumulated over time, in order to ensure that the evolutionary rates of piRNA pathway genes are correctly calculated, the whole genomes of the parental strains, *D. arizonae* (stock number: 15081-1271.17) and *D. m. wrigleyi* (stock number: 15081-1352.22) obtained from UC San Diego Drosophila Stock Center, were sequenced using both paired-end and mate pairs (8kb insert). De novo genome assemblies were performed in two steps: first paired-end reads were assembled in contigs using IDBA [47], and then pre-assembled contigs were scaffolded based on mate-pairs data using SSPACE [48].

To detect signatures of selection in genes involved in the primary and secondary piRNA biogenesis, the coding sequences of the *D. arizonae* and *D. m. wrigleyi* were obtained using the CDS of the *D. mojavensis* r1.04 genome as a reference. The recovered coding sequences of the parental lines were aligned using MAFFT [49]. Next, pairwise tests of selection (*ω* = ratios of nonsynonymous (d_N_)/synonymous (d_S_) nucleotide substitution) and positive selection across sites were computed using CODEML, PAML 4.9 [50]. The codon-based analysis was performed using an F3x4 codon matrix, fixed branch lengths, and alpha values by comparing two pairs of site-specific models: M1a (nearly neutral) vs. M2a (positive selection) and M7 and M8. M1a assumes two categories of site classes: sites with d_N_/d_S_ < 1 (negative selection) and sites with d_N_/d_S_ = 1 (neutral evolution) and M2a assumes three categories of sites: sites with d_N_/d_S_ < 1 (negative selection), sites with d_N_/d_S_ = 1 (neutral evolution) and sites with d_N_/d_S_ > 1 (positive selection). M7: assumes ten categories following a beta-distribution of sites, all with different d_N_/d_S_ ≤ 1and M8: assumes ten categories following a beta-distribution of sites, grouped, all with different d_N_/d_S_ < 1, and an additional 11th category with d_N_/d_S_ > 1 (positive selection allowed). Each pair of models is compared using a likelihood ratio test (LRT). Rejection of M1 in favor of M2a and M7 in favor of M8 indicates positive selection. The posterior probabilities of suggested sites under positive selection were calculated using the Bayes empirical Bayes method (BEB) to calculate the posterior probabilities for sites [51].

### 2.6. Small RNAs and Ping-Pong Analyses

The small RNA-Seq analyses were performed using the method described by Fablet et al. [52]. The data were initially cleaned using Cutadapt [53] to remove the adapter sequences, and PRINSEQ-lite version 0.20.4 [54] was used to filter the reads with a size ranging from 23 to 30 nucleotides to select piRNAs. The TEcount module from TEtools [37] was used to map the sense and antisense piRNA reads against our TE library, previously used differential TE expression analysis. Small RNA read counts were normalized using miRNA sequences of *D. mojavensis* (available at http://52.23.126.124/genomes/Drosophila_mojavensis/dmoj_r1.04_FB2015_02/fasta/dmoj-all-miRNA-r1.04.fasta.gz, accessed on 10 December 2021). For this, miRNA data were aligned, using Bowtie [55], against the cleaned small RNA data (before the size filtering). Expression analyses of piRNAs mapped to TEs were performed in R [46]. For overexpressed TEs previously identified in ovaries and testes, ping-pong signature was analyzed using signature.py pipeline with the options minimum size = 23 and maximum size = 30 [56].

## 3. Results

### 3.1. Differential Expression of TEs in the Comparison D. arizonae vs. D. mojavensis

Analyses of TE expression in parental lines showed that from a total of 298 candidate TE families, 243 (81.2%) were found expressed in ovaries, from which 88% are present in *D. arizonae* and *D. m. wrigleyi* genomes, 9.9% are TE families exclusive to *D. m. wrigleyi,* and 2.1% are exclusive to *D. arizonae*. In testes, a total of 250 (83.5%) TE families were found expressed, of which 86.7% are common to *D. arizonae* and *D. m. wrigleyi*, 11.2% are exclusive to *D. m. wrigleyi,* and 2.1% are exclusive to *D. arizonae*.

**Figure 1:**
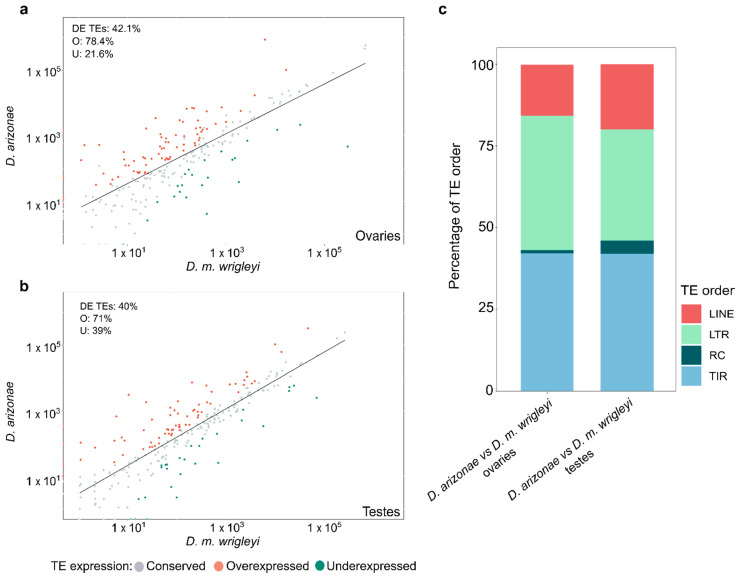
Expression profile of TEs in gonads of *D. arizonae* compared with *D. m. wrigleyi* subspecies. Scatter plots representing differential TE expression in (**a**) ovaries and (**b**) testes between *D. arizonae* and *D. m. wrigleyi.* TE families were considered as DE when they presented Log2(FoldChange) ≥ |1| and *p*-value adjusted (corrected by FDR) < 0.01. (**c**) Percentage of DE TEs families between parental lines classified by TE order. Transposable elements from TIR and RC orders belong to Class II elements, known as DNA transposons. Transposable elements from LTR (long terminal repeat) and LINE (long interspersed nuclear element) orders belong to Class I elements, known as retrotransposons. DE TEs: deregulated TEs; O: overexpressed TEs; U: underexpressed TEs.

Comparative analyses of *D. arizonae* and *D. m*. *wrigleyi* transcriptomes showed that 42.1% (102) of TE families were differentially expressed (DE) in ovaries, whereas 40% (100) of the TE families were DE in testes (Figure 1a,b). The expression profiles in the parental species showed a bias in the distribution of fold changes toward TE overexpression in *D. arizonae* ovaries and testes compared with *D. m. wrigleyi* (*X*^2^ = 31.853, *p* = 1.663 × 10^−8^; *X*^2^ = 16.81; *p* = 4.132 × 10^−^^5^) (Figure 1a,b). Among the DE TE families in both reproductive tissues, LTR (long terminal repeats) and TIRs (terminal inverted repeats) elements were overrepresented in both comparisons (Figure 1c).

### 3.2. TE Expression in the Hybrid Germlines Compared with the Parental Lines

**Figure 2:**
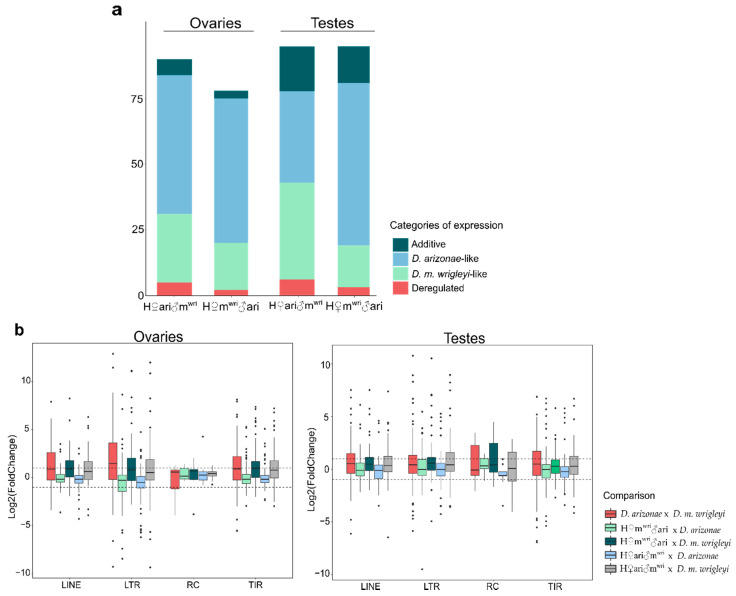
TE expression in hybrids gonads. (**a**) The total number of TE families in hybrid ovaries and testes classified in different categories of expression. (**b**) Global expression of TEs orders according to Log2(FoldChange), between parental lines and between hybrids and parental lines. Dotted lines represent Log2(FoldChange) > |1| and *p*-value adjusted < 0.05.

In order to better understand the TE dynamics in the hybrid genome, we have classified the expression in four different categories, (1) *D. arizonae*-like expression: TE families showing expression profile more similar to *D. arizonae*, (2) *D. m. wrigleyi*-like expression: TE families exhibiting expression profile more similar to *D. m. wrigleyi*, (3) Additive expression: TE families presenting intermediate expression between both parental species, and (4) Deregulated expression: TE families found over or underexpressed in hybrids in comparison to both parental lines. According to these criteria, we observed that in ovaries, most of the DE TE families exhibited *D. arizonae*-like expression (H♀m^wri^♂ari: 53 (58.8%) and H♀ari♂m^wri^: 55 (70.5%), followed by *D. m. wrigleyi*-like expression (H♀m^wri^♂ari: 26 (28.8%) and H♀ari♂m^wri^: 18 (23%)), additive expression (H♀m^wri^♂ari: 6 (6.66%) and H♀ari♂m^wri^: 3 (3.8%)) and deregulated expression (H♀m^wri^♂ari: 5 (5.5%) and H♀ari♂m^wri^: 2 (2.5%)) (Figure 2a, Table S1). In testes, most of the DE TE were classified into the categories *D. arizonae*-like and *D. m. wrigleyi*-like expression. However, in this gonadal tissue, the expression profile was influenced by the cross direction, differently from the results observed in ovaries. In H♀m^wri^♂ari males, most of the DE TE were classified as *D. m. wrilgeyi*-like expression (35–38.9%), followed by *D. arizonae*-like (35–36.8%), Additive expression (17–17.8%) and Deregulated expression (6–6.3%). In the male reciprocal hybrid, H♀ari♂m^wri^, we observed that the category of the expression containing most of TE was *D. arizonae*-like expression (62–65.2%), followed by *D. m. wrigleyi*-like expression (16–16.8%), additive expression (14–14.7%) and deregulated expression (3–3.1%) (Figure 2a, Table S2).

In the female gonads of H♀m^wri^♂ari, the five TE families deregulated were classified as RC (*Helitron-5*), TIR (*REP-2, BARI-1*), and LTR (*Gypsy-6* and *Copia-29*). Interestingly, only the retrotransposon *Copia-29* was also DE between the parental lines, and all TE families, except *Helitron-5*, were common to *D. arizonae* and *D. m. wrigleyi* genomes. In H♀ari♂m^wri^ ovaries, the two deregulated TEs were *BARI-1*/TIR (such as H♀m^wri^♂ari) and *Copia-3*/LTR (DE in between parental lines), both present in the parental genomes. The deregulated TEs found in H♀m^wri^♂ari testes were classified as LTR (*Copia-29, Gypsy-14, Gypsy-30, Gypsy-35*) and RC (*Helitron-2*) elements. Moreover, two of these TEs (*Gypsy-35* and *Helitron-2*) were also deregulated between parental lines, and only the *Helitron-2* family was exclusive to *D. m. wrigleyi* parental genome. In testes of H♀ari♂m^wri^, the three TE families were classified as TIR (*piggyBac-3*) and LINE (*Helena* and *R2*, which were also deregulated between parental species), all of them present in both parental genomes.

Considering the global TE expression in hybrids vs. parental lines, we observed a wide range of expression in ovaries and testes, mainly for LTRs and TIRs elements, which presented the most extreme Log2(FoldChange) (Figure 2b). Comparing the hybrid expression related to parental lines, it is worth noting differences in the degree of expression according to cross direction and the parental line, since LINE (Log2(FoldChange) mean: 1.18), LTR (Log2(FoldChange) mean: 1.28) and TIR (Log2(FoldChange) mean: 1.24) orders display a bias for overexpression in H♀m^wri^♂ari females compared with *D. m. wrigleyi* parent (Figure 2b), which is also observed in H♀ari♂m^wri^ female gonads (Log2(FoldChange) mean: 0.94, 0.99 and 1.19 for LINE, LTR, and TIR orders, respectively). In testes, a similar pattern is observed in hybrids of the same cross direction (H♀ari♂m^wri^) but with RC order (Figure 2b).

**Figure 3:**
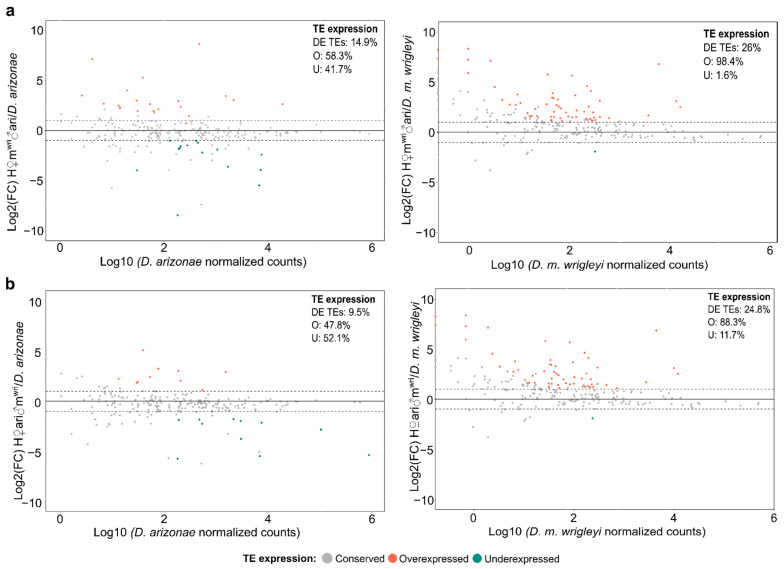
TE expression in ovaries of hybrids vs. parental lines. (**a**) log2 of the ratio (counts in H♀m^wri^♂ari/counts in parental lines (*D. arizonae* and *D. m. wrigleyi*)); (**b**) log2 of the ratio (counts in H♀ari♂m^wri^/counts in parental lines (*D. arizonae* and *D. m. wrigleyi*)). Dotted lines represent Log2(FoldChange) > |1| and *p*-value adjusted < 0.05. DE TEs: deregulated TEs; O: overexpressed TEs; U: underexpressed TEs.

Regarding the expression profile of TEs in ovaries, we observed that H♀m^wri^♂ari and H♀ari♂m^wri^ showed 26% (63) and 24.8% (60) DE TEs compared with *D. m. wrigleyi,* while compared with *D. arizonae* they presented 14.9% (36) and 9.5% (23) of DE TEs. Interestingly, the expression profile of reciprocal hybrids compared with *D. m. wrigleyi* also evidenced a bias toward TE overexpression (H♀m^wri^♂ari vs. *D. m. wrigleyi*: *X*^2^ = 57.143, *p* = 4.053 × 10^−14^; H♀ari♂m^wri^ vs. *D. m. wrigleyi: X*^2^ = 36.817, *p* = 1.298 × 10^−9^) (Figure 3a,b).

**Figure 4:**
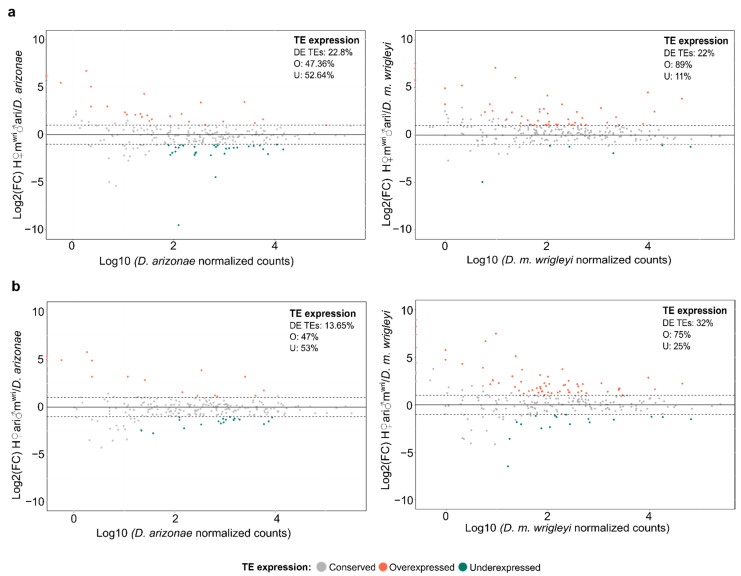
TE expression in testes of hybrids vs. parental lines. (**a**) log2 of the ratio (counts in H♀m^wri^♂ari/counts in parental lines (*D. arizonae* and *D. m. wrigleyi*)); (**b**) log2 of the ratio (counts in H♀ari♂m^wri^/counts in parental lines (*D. arizonae* and *D. m. wrigleyi*)). Dotted lines represent Log2(FoldChange) > |1| and *p*-value adjusted < 0.05. DE TEs: deregulated TEs; O: overexpressed TEs; U: underexpressed TEs.

In testes of reciprocal hybrids, the proportion of DE TEs found in H♀m^wri^♂ari male gonads in relation to the parental lines was very similar (~22%) (Figure 4a). However, H♀ari♂m^wri^ showed 32% (80) of DE TEs in relation to *D. m. wrigleyi* and 13.65% (34) compared with *D. arizonae* (Figure 4b). Moreover, like in ovaries, the expression profile of TE in hybrids presented a bias toward overexpression only when compared with *D. m. wrigleyi* (H♀m^wri^♂ari vs. *D. m. wrigleyi*: *X*^2^ = 32.073. *p* = 1.485 × 10^−8^; H♀ari♂m^wri^ vs. *D. m. wrigleyi:* *X*^2^ = 19.012, *p* = 1.299 × 10^−5^).

### 3.3. piRNA Repertoire in Female and Male Hybrid Germlines

**Figure 5:**
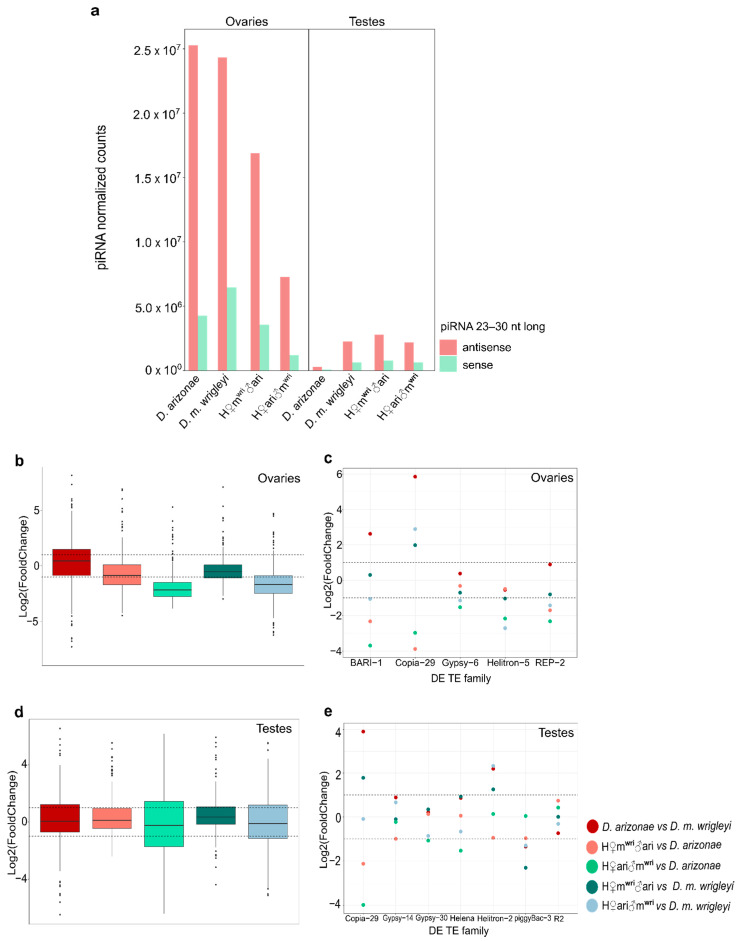
Small RNA abundance in gonads of hybrid and parental lines. (**a**) TE-derived small RNA production for ovaries (left) and testes (right) of *D. arizonae*, *D. m. wrigleyi*, and their reciprocal hybrids; (**b**) TE-derived 23 to 30 nt small RNA modulation upon interspecific hybridization in female gonadal tissues; (**c**) TE-specific piRNA abundance for upregulated TEs found in ovaries; (**d**) TE-derived 23 to 30 nt small RNA modulation upon interspecific hybridization in male gonadal tissues; (**e**) TE-specific piRNA abundance for upregulated TEs identified in testes. Small RNA amounts were normalized relative to miRNAs. Dotted lines represent Log2(FoldChange) = |1|.

The analyses of piRNAs allowed us to identify larger amounts of TE-derived regulatory piRNA populations in ovaries than in testes in parental lines and reciprocal hybrids *(*Figure 5a). Moreover, when comparing the parental lines in ovaries, we found 152 TE families showed up to a 2-fold difference in the amount of TE-derived piRNAs, of which 35% presented a higher amount of piRNAs in *D. arizonae.* As shown in Figure 5b, the majority of piRNAs mapping to TEs presented similar expression in the female parental lines (Log2(FoldChange) mean: 0.26). In the female reciprocal hybrids, the global abundance of TE-derived piRNAs was lower when compared with the parental lines (Figure 5b) (H♀m^wri^♂ari vs. *D. arizonae* = Log2(FoldChange) mean: −0.58; H♀m^wri^♂ari vs. *D. m. wrigleyi* = Log2(FoldChange) mean: −0.32; H♀ari♂m^wri^ vs. *D. arizonae* = Log2(FoldChange) mean: −1.8; H♀ari♂m^wri^ vs. *D. m. wrigleyi* = Log2(FoldChange) mean: −1.6). However, it is noteworthy that despite the lower amount of TE-derived piRNAs identified, very few TEs families were upregulated.

**Figure 6:**
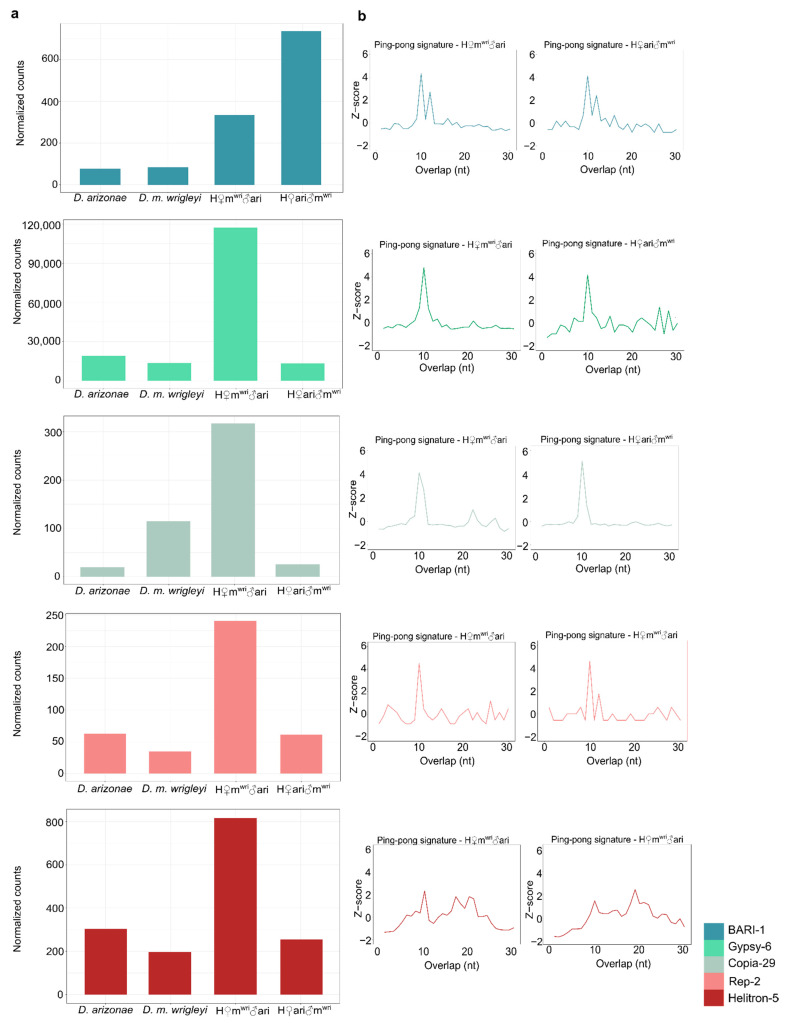
TE transcripts and TE-derived piRNAs in females assessed from RNA-Seq. (**a**) Expression level of TEs found upregulated in H♀m^wri^♂ari and H♀ari♂m^wri^ ovaries. (**b**) Ping-pong signatures for 23–30 nt RNAs in *D.* H♀m^wri^♂ari and H♀ari♂m^wri^ ovaries. Significant enrichment in 10-nt overlaps (i.e., ping-pong signatures) is considered when *z*-score > 2.58.

To better understand the regulation process of the DE TEs, we analyzed the amount of piRNAs mapping to these specific TEs and the presence of ping-pong signature, which can indicate the secondary piRNA biogenesis activation. Our results showed a two-fold decrease compared with at least one parental line for *BARI-1, Copia-29, Gypsy-6, Helitron-5* and *REP-2*-specific piRNAs in H♀m^wri^♂ari females, which can contribute to a less efficient post-transcriptionally regulation mechanism, and hence, the overexpression of these TE families (Figure 5c and Figure 6a). Ping-pong signature was observed for all these TE families, except *Helitron-5* (Figure 6b). In H♀m^wri^♂ari, which the maternal line is *D. m. wrigleyi*, we observed a greater difference between the amounts of TE-specific piRNAs in relation to *D. arizonae*, the paternal line (Figure 5c). A similar pattern was observed in H♀ari♂m^wri^ since only *BARI-1* was upregulated. As displayed in Figure 5b, the abundance of *BARI-1*-specific piRNAs was two-fold smaller than in both parental lines, which likely contributed to its overexpression. The only exception to this pattern was the *Helitron-5* family. Once the amount of *Helitron-5*-specific piRNAs was found to be similar to both parental lines (Figure 5c), however, it was not able to control its expression, which can be observed by lack of ping-pong signature (Figure 6b).

**Figure 7:**
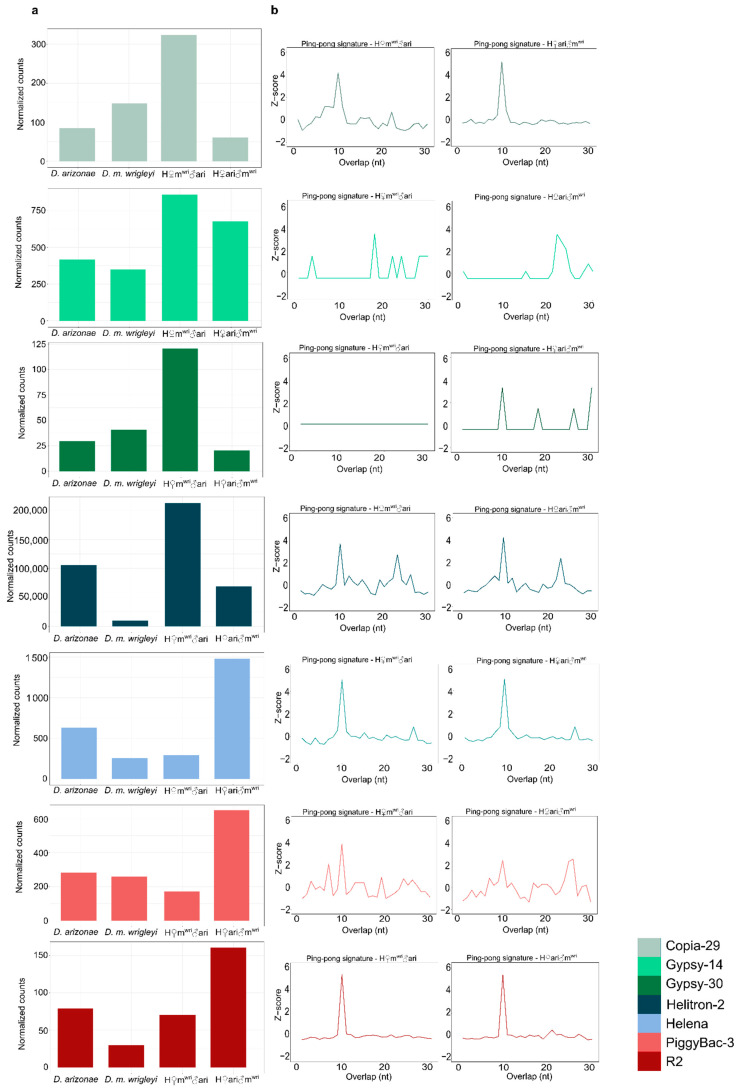
TE transcripts and TE-derived piRNAs in males assessed from RNA-Seq. (**a**) Expression level of TEs found upregulated in H♀m^wri^♂ari and H♀ari♂m^wri^ testes. (**b**) Ping-pong signatures for 23–30 nt RNAs in *D.* H♀m^wri^♂ari and H♀ari♂m^wri^ testes. Significant enrichment in 10-nt overlaps (i.e., *ping-pong* signatures) is considered when *z*-score > 2.58.

Overall, piRNA abundance greater than 2-fold was observed for 126 TE families compared to *D. arizonae* and *D. m. wrigleyi* males. From these, 27.7% showed piRNA abundance greater than two-fold and 20.7% smaller than two-fold of difference; most of the TE-derived piRNAs presented similar amounts in both parental males, as shown in Figure 5d (Log2(FoldChange) mean: 0.09). Differently from the results observed for ovaries, the number of piRNAs in the hybrid testes was not very variable; thus, most of the TE-derived piRNAs abundance was similar to parental lines (H♀m^wri^♂ari vs. *D. arizonae* = Log2(FoldChange) mean: 0.3; H♀m^wri^♂ari vs. *D. m. wrigleyi* = Log2(FoldChange) mean: 0.4; H♀ari♂m^wri^ vs. *D. arizonae* = Log2(FoldChange) mean: −0.12; H♀ari♂m^wri^ vs. *D. m. wrigleyi* = Log2(FoldChange) mean: −0.02) (Figure 5d). Likewise, for most of the upregulated TEs in testes, the amounts of complementary piRNAs were not different from the parental lines (Figure 5e). Furthermore, the presence of secondary piRNA biogenesis, observed for most of the upregulated TEs (Figure 7a,b), may not be the main mechanism of TE regulation in testes, as it is in ovaries of *Drosophila*.

### 3.4. Divergence of Expression and Selective Process Acting on piRNA Pathway Genes

**Figure 8:**
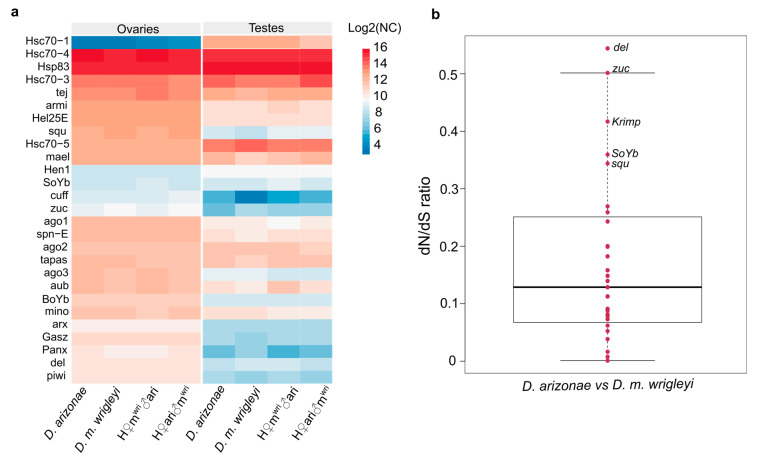
piRNA pathway genes that act in the biogenesis of primary and secondary via piRNAs. (**a**) Heatmap representing gene expression in ovaries (left) and testes (right) of *D. arizonae*, *D. m. wrigleyi,* H♀m^wri^♂ari and in H♀ari♂m^wri^. Colors indicate the level of expression according to log2 of normalized counts (NC). (**b**) d_N_/d_S_ ratio for the 33 genes involved in the piRNA pathway between parental species. The genes under negative relaxed selection (d_N_/d_S_ > 0.3) for each comparison are identified in the plot.

We performed gene expression analyses to detect if the overexpression of specific TEs was associated with the divergence of piRNA pathway genes. Our results showed that the expression of several piRNA genes is quite conserved among the female gonads of hybrids and parental lines (Figure 8a). However, considering different gonadal tissues, the expression level is quite distinct (Figure 8a, Tables S2 and S3).

More specifically, in female reproductive tissues, no genes participating in the primary and secondary piRNA pathways were DE, either between the parental species (*D. arizonae* x *D. m. wrigleyi*) or between hybrids and their parental lines (Figure 8a, Table S3). In parental testes, most of the genes involved in the piRNA pathway presented conserved expression, except for the genes *Cubitus interruptus (ci),Methyltransferase 2 (Mt2) and Zucchini (zuc)* (Figure 8a, Table S4). In H♀m^wri^♂ari testes, the differential expression of the gene *zuc* was detected when compared with *D. arizonae* and the genes *Aubergine* (*aub), ci, Cutoff (cuff), Mt2,* and *Panoramix* (*Panx)* were DE in relation to *D. m. wrigleyi*. In the testes of the reciprocal hybrids, H♀ari♂m^wri,^ we identified underexpression of *Heat shock protein cognate 1* *(Hsc70–1)* in relation to both parental lines and of the genes *ci, cuff, Heat shock 70-kDa protein cognate 3 (Hsc70–3)*, and *Mt2* were DE in comparison with *D. m. wrigleyi* (Figure 8a, Table S4).

To further investigate the causes leading to differential expression of some piRNA genes between hybrids and parental lines, selective tests were performed. The analyses were carried out for 28 genes involved in the piRNA pathway, as shown in Figure 8b (Table S4). Firstly, we performed pairwise ratio tests and found no signal of positive selection between *D. arizonae* vs. *D. m. wrigleyi* (Figure 8b). Most of the genes analyzed showed d_N_/d_S_ ratios ranging from 0 to 0.25 in the comparisons *D. arizonae* and *D. m. wrigleyi*. Moreover, we found signals of relaxed negative selection (*ω* > 0.3) for several genes in each comparison (Figure 8b, Table S5), such as *Deadlock* (*del),Krimper (krimp), Sister of Yb (SoYb), squash (squ),* and *zuc*, indicating an ongoing divergence process (Figure 8b). Likelihood logs used for LRT determination showed positive selection, with statistics support, only for the gene *del* (Table S5) when comparing the models M1a and M2a, and M7 and M8 (showing two sites under positive selection). Both models showed greater 2∆ln*L* than critical values from a *χ*^2^ distribution with degree of freedom = 2 (*p* < 0.02). Although without statistics support for positive selection, likely due to the recent divergence time, several genes showed positively selected sites under the models M2a and M8. Among these genes, we found *Armitage* (*armi)*, *aub, ci, cuff, Minotaur (mino), Mt2, SoYb,* Shutdown (*shu)*, *Tapas*, *Tejas* (*tej)*, *Vreteno* (*vret)*, and *zuc* (Table S5), several of the which displayed signals of relaxed negative selection, corroborating their initial divergence process.

## 4. Discussion

The profile of TE expression in the sibling species *D. arizonae* and *D. m. wrigleyi* showed that ovaries and testes have similar rates of DE TEs, which was also observed in *D. buzzatii* and *D. koepferae* [20]. Regarding the DE TEs in ovaries and testes of parental lines, an overrepresentation of LTR (Long Terminal Repeats) and TIRs was observed, likely due to greater proportions of these TE orders in *D. arizonae* and *D. mojavensis* genomes, as reported by *Drosophila* 12 Genomes et al. [57] and Rius et al. [58] for *D. mojavensis*. In this study, TE differential expression in ovaries of *D. arizonae* and *D. m. wrigleyi* was found higher than the rate observed in crosses between *D. arizonae* and another *D. mojavensis* subspecies [34]. This fact may be linked to genetic variability and TE content of different *D. mojavensis* subspecies. The strain used in this study derives from a well-structured population, endemic from Santa Catalina Island, which exhibits significant genetic differentiation from all the three other *D. mojavensis* subspecies [59]. Thus, the evolutionary history of the *mojavensis* species group could have contributed to the accumulation of genetic divergence in TE copies and TE content across different strains, as observed in other *Drosophila* species [60]. However, such differences could also be due to the TE library used to annotate the expressed repeats, which differed in the two cases. Lopez-Maestre et al. [34] used a TE library-based only in the *D. mojavensis* genomes, while in this study, our TE library was composed of TE sequences from *D. mojavensis* and *D. arizonae* genomes, which could influence the results of differential expression observed.

Comparing TE expression of hybrids with both parental lines, we observed very few deregulated TE families (H♀m^wri^♂ari: 2% and 2%; H♀ari♂m^wri^: 0.8% and 1.2%, for ovaries and testes, respectively). These findings concur with the results reported by Lopez-Maestre et al. [34]. However, it is quite different from the findings of Romero-Soriano et al. [20] for D. *buzzatii-D. koepferae* (5.1%) and Kelleher et al. [9] for *D. melanogaster-D. simulans* hybrids (12.1%). These results suggest that longer divergence time of two genomes lead to a greater rate of TE deregulation, thus in species recently diverged, such as *D. arizonae* and *D. m. wrigleyi* (~1.5 million years [29]), the genetic divergence in TE copies will be smaller, and consequently, the TE deregulation in their reciprocal hybrids. Additionally, we have observed a bias toward TE upregulation in ovaries and testes compared with the parental lines, as well as distinct levels of expression of different TE orders in male and female gonads, which was also reported in *D. buzzatii-D. koepferae* hybrids [20]. Hence, we sequenced and analyzed the piRNA pool of parental species and their respective hybrids to understand the factors underlying the deregulation of some TE families in interspecific hybrids of *D. arizonae* and *D. m. wrigleyi.*

Globally, we showed that ovaries produce more piRNAs derived from TEs than testes, which was also observed in *D. melanogaster* and *D. simulans* [61]. In hybrid ovaries, five TE families (*BARI-1, Copia-29*, *Gypsy-6*, *REP-2*, and *Helitron-5*) were found upregulated in H♀m^wri^♂ari and only one in H♀ari♂m^wri^ (*BARI-1*). These results can be explained by the inheritance of different TE copies from the parental genomes. *BARI-1*, *Copia-29*, *Gypsy-6*,1, and *REP-2* are elements present in both parental genomes, and hence different copies, with accumulated divergence, are inherited by the hybrids. Moreover, in H♀m^wri^♂ari, a decrease in TE-specific piRNAs in relation to *D. arizonae*, the paternal line, was observed. This could indicate that divergence of TE copies from different genomes can influence the TE upregulation in female hybrids. Probably, the observed ping-pong signature reflects activation of the secondary piRNA biogenesis pathway, but it was not enough to silence all the TE copies due to divergence of sequences. Likewise, the abundance of BARI-1-specific piRNAs in H♀ari♂m^wri^ ovaries was more than two-fold smaller than in both parental lines, contributing to the regulatory mechanism’s failures. Similar results have been reported in the literature for the LTR retrotransposon *Frogger* in *D. arizonae-D. m. mojavensis* female hybrids [34]. As observed here, *Frogger* was upregulated even if presenting strong ping-pong amplification due to the diversity of copies expressed in the parental genome, which limits the capacity to attribute transcripts and piRNAs to specific insertions [34]. In the same way, it was observed that in female hybrids of *D. melanogaster-D. simulans* and *D. buzzatii-D. koepferae,* TE derepression is not always associated with disrupting the *ping-pong* cycle [9,20].

In hybrids from more divergent species, the failure to regulate TEs is widely associated with adaptive divergence in piRNA pathway genes [9,20]. However, divergence in piRNA pathway genes does not explain the TE derepression in female hybrids from more recently diverged species, such as *D. arizonae* and *D. m. wrigleyi*, since no differential expression was observed, although some of the piRNA genes were under relaxed negative selection, indicating that the divergence process is ongoing, but that a strong sequence divergence has not yet been achieved. Therefore, in female hybrids from *D. arizonae* and *D. m. wrigleyi* crosses, it seems that differences in TE content of the parental lines are the main players in TE deregulation, which is often observed in *Drosophila*, for different populations of the same species or different species [51,53,62]. It is important to point out that other factors may be involved in this phenomenon, such as epigenetic changes and failure of the transcriptional regulatory mechanism. In *Drosophila* ovaries, the Piwi protein plays a major role in heterochromatin modification by interacting with several proteins, leading to heterochromatin formation [61,62,63,64]. Despite *D. melanogaster-D. simulans* hybrids have normal amounts of piRNAs; their heterochromatin genes are downregulated, which is associated with *Hybrid male rescue* (*Hmr*) and *Lethal male rescue* (*Lmr*) divergence between the parental lines, exerting an effect on heterochromatin genes [18,65,66]. Nevertheless, our recent study demonstrated that female hybrids from *D. arizonae* × *D. m. wrigleyi* have very few deregulated genes, most of them overexpressed and with a distinct function, which was not related to heterochromatin formation [30]. Our findings suggest that in ovaries of H♀m^wri^♂ari and H♀ari♂m^wri^ heterochromatin genes are not responsible for failures in TE derepression.

Regarding TE regulation in male hybrids, we report that most of the DE TEs are overexpressed, in contrast to the results observed by Romero-Soriano et al. [20], who described underexpression of TEs in hybrid males from *D. buzzatii* and *D. koepferae*. We show that the TE regulation in male gonads may be different from the mechanisms in female germline since less abundance of TE-derived piRNAs was detected. Furthermore, we observed that TE-derived piRNAs in hybrids are similar to parental lines, even considering the upregulated TEs. Moreover, the TE derepression observed, despite the detection of ping-pong amplification and the presence of sense and antisense TE-mapping piRNAs, suggests that there may be other more efficient mechanisms underlying TE regulation in testes. Considering this result, we cannot consider that, like in ovaries, the divergence of TE copies and lack of piRNA mapping TEs are the only features influencing TE regulation in male gonads. Indeed, in *Drosophila* testes, the piRNA pathway functions are different from in ovaries [67], and it may be related to a specific developmental stage and degree of hybrid sterility [68]. According to Quenerch’du et al. [69], different populations of piRNA are present in the spermatogonia or primary spermatocytes. In spermatogonia, piRNAs are predominantly transposon-mapping piRNAs, and the ping-pong signature has been verified for several of these sequences. However, in testes enriched in primary spermatocytes, *Ago3* is not expressed, but the ping-pong signature is observed, indicating that a noncanonical ping-pong cycle functions in spermatogenesis. In our analyses of piRNA-related genes, no differential expression or absence of Ago3 was observed in testes; nevertheless, several other important genes acting in the piRNA pathway were differentially expressed, such as *aub, ci, cuff, mt2, Panx,* and *zuc*. *aub* is a gene that encodes for an RNA-binding protein of the Piwi clade, which is able to silence TEs in the germline [70,71,72]. The *cuff* gene has an essential role in piRNA production from dual-strand clusters [73]. The gene *ci* is a transcription factor required to activate the transcription of single-strand clusters in somatic follicle cells of fly gonads, such as the *Flamenco* locus [74,75]. The gene *Mt2* is responsible for silencing retrotransposons in *Drosophila* somatic cells through the initiation of histone H4K20 trimethylation [76]. *Panx* can enforce transcriptional silencing by binding to the target-engaged Piwi-piRNA complex [77,78]. On the other hand, the gene *zuc* is necessary for the biogenesis of Piwi-bound piRNAs [79]. The differential expression of these genes in male hybrids might affect the piRNA silencing mechanism to some extent, as was reported in other studies [20,34]. Additionally, the ongoing divergence process acting on these piRNA genes, here identified as signals of relaxed negative selection and possible positive selection acting on a few sites, can have a stronger effect in males than in females. Banho et al. [30] showed that H♀m^wri^♂ari and H♀ari♂m^wri^ males display more differentially expressed genes than females, likely due to specific epistatic factors from sexual chromosomes and autosomes.

Like ovaries, we cannot exclude other factors that play a potential role in TE derepression in testes. In fact, the decreased expression of a heat shock protein, Hsp70-1, observed in the hybrid male gonads could influence TE deregulation. Proteins of this family have been shown to play roles in the siRNA, miRNA, and piRNA regulatory pathways [80,81,82]. The Hsp70 chaperone has recently been shown to interact with components of chaperone machinery involved in piRNA biogenesis, and the disruption of these proteins decreases the efficacy of TE repression [80]. Therefore, in testes, the post-transcriptional and transcriptional silencing mechanisms are likely more complex and prevent TE derepression, which in hybrid testes is disturbed due to gene differential expression and regulatory incompatibilities.

## 5. Conclusions

The findings of this study bring several contributions to the current literature. We show that the TE deregulation observed in female hybrids of *D. arizonae* and *D. m. wrigleyi* is likely associated with fewer complementary piRNAs in relation to one parental line. These findings indicate that differences in TE content and divergence of TE copies play an important role in the post-transcriptionally regulation mechanisms in ovaries. However, in testes, smaller production of piRNAs was detected, indicating that in this tissue, these small RNAs may not be the main regulatory mechanisms to control TE repression, as in ovaries. Moreover, in testes, differentially expressed genes presenting post-transcriptional and transcriptional silencing functions may be involved in the less effective TE repression.

## Data Availability

The data sets used and/or analyzed during the current study are at https://www.ncbi.nlm.nih.gov/sra (accessed on 10 December 2021), submissions PRJNA691040 and PRJNA783761.

## Supplementary Materials

The following are available online at https://www.mdpi.com/article/10.3390/cells10123574/s1, Table S1: Expression level of TE families identified in hybrid ovaries and their respective inheritance profile, Table S2: Expression level of TE families identified in hybrid testes and their respective inheritance profile, Table S3: Expression level of genes acting on piRNA pathway and chromatin modification in ovaries of *D. arizonae*, *D. m. wrigleyi* and their reciprocal hybrids, Table S4: Expression level of genes acting on piRNA pathway and chromatin modification in testes of *D. arizonae, D. m. wrigleyi* and their reciprocal hybrids, Table S5: Evolutionary rate of piRNA genes determined by d_N_/d_S_ ratio and site evolution.
